# Synthesis, Electrochemistry, and Optoelectronic Properties of Biphenyl-EDOT-Based Electrochromic Polymers

**DOI:** 10.3390/nano15211643

**Published:** 2025-10-28

**Authors:** Shuanglai Shen, Yaoteng Deng, Daize Mo, Mengze Xu, Kuirong Deng

**Affiliations:** 1 School of Applied Physics and Materials, Wuyi University, Jiangmen 529020, China; 15171640208@163.com (S.S.); dengyaoteng@163.com (Y.D.); 2 Department of Biology, Faculty of Arts and Sciences, Beijing Normal University, Zhuhai 519087, China

**Keywords:** biphenyl, electrochromic, EDOT

## Abstract

In this study, two novel hybrid monomers (**4BD-EDOT** and **3BD-EDOT**) containing a biphenyl group and a 3,4-ethylenedioxythiophene (EDOT) unit were synthesized and polymerized electrochemically in a CH_2_Cl_2_-Bu_4_NPF_6_ electrolyte solution. Characterizations of the resulting **P4BD-EDOT** and **P3BD-EDOT** were studied by CV, scanning electron microscopy (SEM), and spectroelectrochemistry in order to examine the effect of different substitution positions of biphenyl on the electrochromic performance of the resultant hybrid polymers. Both polymers have favorable redox activity (a distinct redox peak) and good redox stability (55–49% electroactivity was retained after 1000 cycles). The spectro-electrochemistry study found that both show a distinct color change from reddish brown to blue/purple for **P4BD-EDOT** with a lower band gap (1.54 eV) and from transparent color to light blue for **P3BD-EDOT** with a larger band gap (1.73 eV). These electrochromic polymer films also have fast switching speed (0.5–0.2 s), with the favorable optical contrast (22.6% at 1100 nm for **P4BD-EDOT**) and decent coloration efficiency (250.4 cm^2^ C^−1^ at 780 nm for **P3BD-EDOT**). All these results show that both monomers have important values related to the electrochromic field. This work also shows that the different substitution positions of the biphenyl unit affect the spectroelectrochemistry and electrochromic characteristics of the resultant hybrid polymers.

## 1. Introduction

In recent years, conductive polymers (CPs) have gained great importance in academic and commercial applications due to their unique advantages and wide range of applications [1,2,3,4]. Oligomers are one of the most widely studied of functional π-conjugated systems, which have received increasing attention due to their ease of synthesis and effectiveness, as well as their applications in various organic devices, including organic electroluminescent diodes (OLEDs) [1], organic photovoltaic devices (OPVs) [2], and electrochromic devices. Among the CPs (polypyrrole, polyaniline, polythiophene, etc.), the polymers based on thiophene units, such as thiophene, bithiophene (BT), thieno[3,2-b]thiophene, and benzo[1,2-b:4,5-b′]dithiophene [3,4], etc., have been greatly developed.

On this basis, the benzene ring is a common unit, which has the advantages of low cost and easy availability. Biphenyls are neutral molecules without a functional group; hence, they must be functionalized prior to use [5]. Biphenyls consist of two benzene rings linked at the 1,1′-position and react similarly to benzene groups, as they both can undergo electrophilic substitution reactions. Biphenyl derivatives, which are also used to produce an extensive range of drugs, products for agriculture, fluorescent layers in OLEDs, and building blocks for basic liquid crystals, are significant intermediates in organic chemistry besides being the structural moiety of an extensive range of compounds with pharmacological activities [6,7,8]. The electrochemical and optical properties of biphenyl-bridged dicarbazole oligomer films were realized by SiddikIcli and co-workers [9]. The polymer shows reversible electrochemical oxidation and strong color changes, and the corresponding ECD devices showed a better redox stability test. Yang et al. [10] also reported a novel BDF core-based copolymer, PBDFTz-SBP, which is decorated with two 2D extended biphenyl side chains. The PBDFTz-based device achieved an excellent photovoltaic performance. But, up to now, BD-based organic conducting polymers have been rarely reported in the field of electrochromics [11,12,13]. On the other hand, the 3,4-ethylenedioxythiophene (EDOT) unit has also received considerable attention as a building block for the synthesis of functional π-conjugated systems in the past decades [14,15,16,17,18,19]. The incorporation of EDOT molecules has been shown to provide unique properties such as a smaller intrinsic band gap of the polymers [20], greater π-donor capacity of tetrathiafulvalene analogs [21,22], and better electrophilic performance. Therefore, many new EDOT-based copolymers have been prepared and investigated.

Hence, this work focuses on elucidating the “substitution position effect” of the biphenyl unit in biphenyl-EDOT-based conjugated systems. We synthesized two constitutional isomers, 4BD-EDOT and 3BD-EDOT, where the biphenyl core is functionalized at the para- and meta- positions, respectively. The central premise is that the substitution pattern dictates the molecular planarity, which is a primary factor controlling the electronic and optical properties of the resulting electrochromic polymers, P4BD-EDOT and P3BD-EDOT. Our findings confirm a strong correlation between the monomeric isomeric structure and the polymer’s band gap, redox characteristics, and color-switching behavior, providing valuable guidelines for molecular engineering of electrochromic materials.

## 2. Results and Discussion

### 2.1. Synthesis and Characterization

**Scheme 1:**
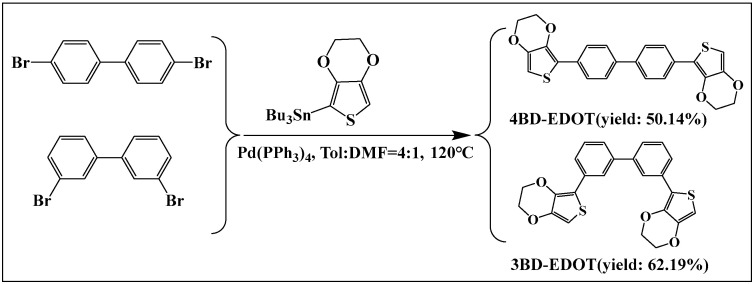
Synthetic routes of **4BD-EDOT** and **3BD-EDOT**.

Scheme 1 outlines the synthetic route to 4BD-EDOT and 3BD-EDOT monomers via Pd-catalyzed Stille cross-coupling of 4,4′/3,3′-dibromobiphenyl with stannylated EDOT, and their corresponding polymers (**P4BD-EDOT** and **P3BD-EDOT**) were prepared by the electrochemical deposition method (Figure S1). The corresponding ^1^H NMR data (Figures S2 and S3), ^13^C NMR spectra (Figures S4 and S5), and IR spectra (Figure S6) are provided in the Supporting Information.

### 2.2. Electrochemical Polymerization of Monomers

**Figure 1:**
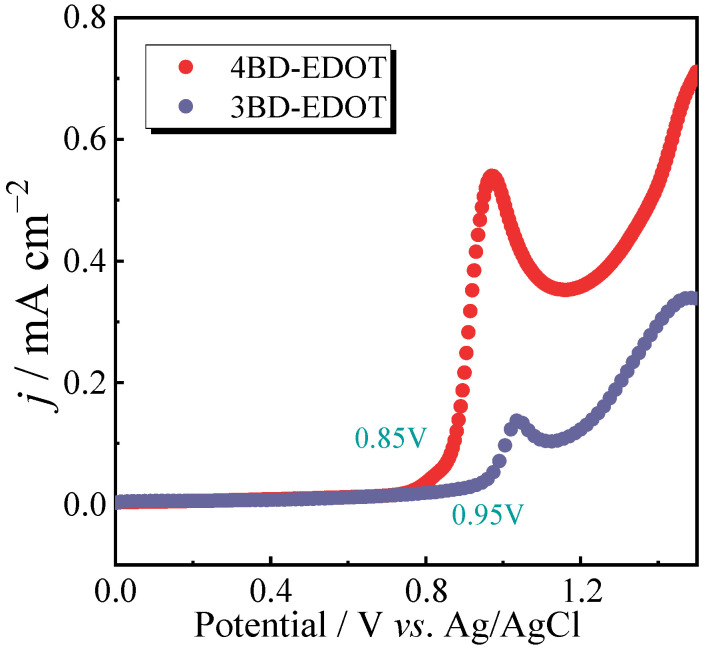
Anodic polarization curves of **3BD-EDOT** and **4BD-EDOT** in 0.1 M CH_2_Cl_2_-Bu_4_NPF_6_ at 50 mV s^−1^.

The anodic polarization curves of **4BD-EDOT** and **3BD-EDOT** were obtained to determine their onset oxidation potential, as shown in Figure 1. The polymerization potential of **3BD-EDOT** is located at 0.95 V, while **4BD-EDOT** shows a lower potential at 0.85 V, which suggests that the **4BD-EDOT** has a high electrochemical activity and can be efficiently polymerized under lower oxidation potential.

**Figure 2:**
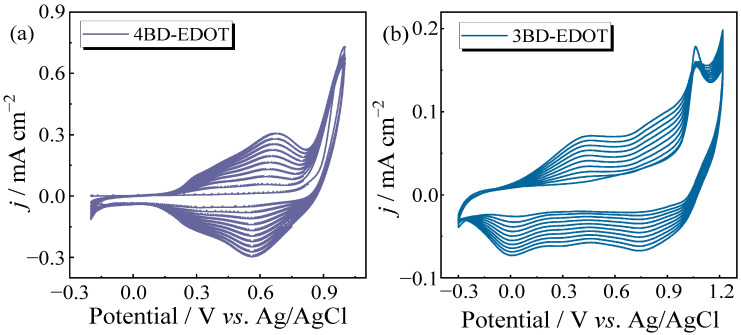
Repeated CV scanning of **4BD-EDOT** (**a**) and **3BD-EDOT** (**b**) with a scan rate of 100 mV s^−1^.

The electrochemical characterization of both monomers was performed by cyclic voltammetry in a dichloromethane/acetonitrile (DCM/ACN) electrolyte solution containing 0.1 M tetrabutylammonium hexafluorophosphate (Bu_4_NPF_6_). The studies were conducted under optimized potential windows: −0.25 V to +1.00 V for **4BD-EDOT** and −0.30 V to +1.20 V for **3BD-EDOT**, with a consistent scan rate of 100 mV·s^−1^ for redox behavior analysis. Figure 2 demonstrates consistent growth of polymer film-associated redox waves with excellent reproducibility during consecutive potential scans. The systematic current density enhancement with cycling, unmarred by anomalous current spikes or irreversible oxidation signatures, confirms the absence of characteristic side reactions, including over-oxidation and polymer chain degradation [23,24]. As shown in Figure 2a, **4BD-EDOT** exhibits a characteristic oxidation peak at 0.67 V with its corresponding reduction peak at 0.55 V, representing a redox couple primarily associated with bi-polaron formation in the polymer backbone. In marked contrast, the cyclic voltametric response of **3BD-EDOT** (Figure 2b) reveals substantially different electrochemical characteristics, displaying two broad redox couples at 0.41/0 V and an additional pair of redox features around 1.06/0.76 V. This multi-stage redox behavior can be attributed to the sequential formation of polarons and bi-polarons within distinct potential windows. Notably, **3BD-EDOT** demonstrates slight electro-polymerization heterogeneity during consecutive scanning cycles, which correlates strongly with the conformation distortion induced by steric hindrance in its molecular architecture. This structural feature likely impedes efficient electron transport along the polymer chains [25].

### 2.3. Electrochemistry of Polymers

**Figure 3:**
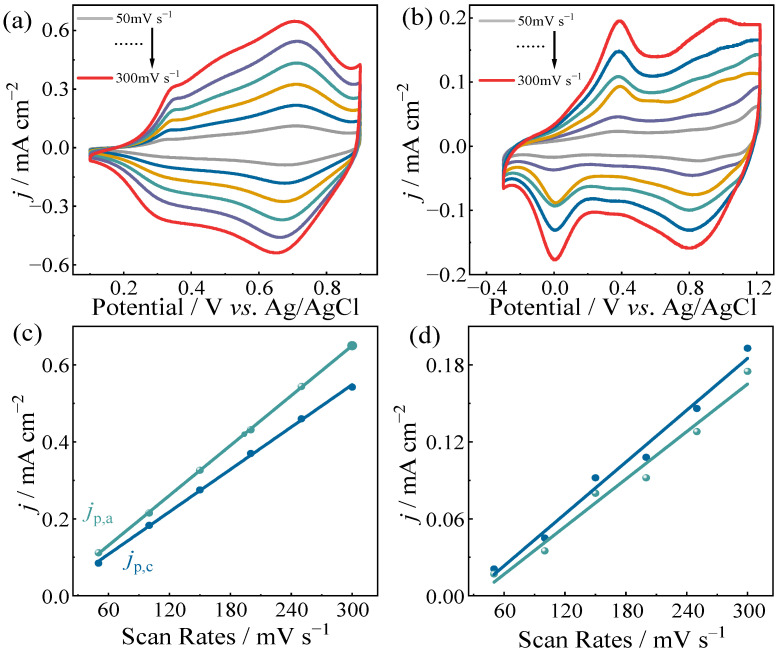
Cyclic voltametric diagrams of (**a**) **P4BD-EDOT** and (**b**) **P3BD-EDOT** at different scan rates (50–300 mV s^−1^) for 3 cycles. The relationship between scan rate and current density of (**c**) **P4D-EDOT** and (**d**) **P3D-EDOT**.

The doping and de-doping behavior of **P4BD-EDOT** and **P3BD-EDOT** were exhibited at different scan rates by the CV method, as shown in Figure 3. Both polymers show well-defined oxidation and reduction peaks with reversible redox processes. However, the shape of the peaks is quite different. The CV curves of **P4BD-EDOT** are irreversible with two pairs of regular redox peaks at 0.34 V/0.29 V and 0.71 V/0.65 V, respectively, and the **P3BD-EDOT**’s redox peaks are apparent and broader (0.38 V/0 V; 1.0 V/0.8 V) (Figure 3b). This presents two redox pairs, which are attributed to its polaron and di-polaron band [26]. The earlier onset of electrochemical activity observed in **P3BD-EDOT** compared to **P4BD-EDOT** can be attributed to its larger inter-ring torsion angle within the biphenyl unit. This stereochemical configuration effectively reduces the ionization potential and optimizes charge distribution, consequently lowering the activation energy barrier for the oxidation process. These combined effects manifest experimentally as a lower onset oxidation potential and premature emergence of redox activity in **P3BD-EDOT** [27]. Interestingly, the higher current intensity of **P4BD-EDOT** shows better redox activity compared to **P3BD-EDOT**. The redox behavior shows that different sites of substituted biphenyls play an important role in regulating the electrochemical properties and may further affect their electrochromic properties. Moreover, with increasing scan rate, the size of the redox peaks becomes more remarkable, while the shape of the peaks has no change, which indicates that these polymers have good stability [28,29]. In addition, the electrochemical processes of the polymers are not diffusion limited according to the high linear relationship between current density and scan rate (Figure 3c,d).

**Figure 4:**
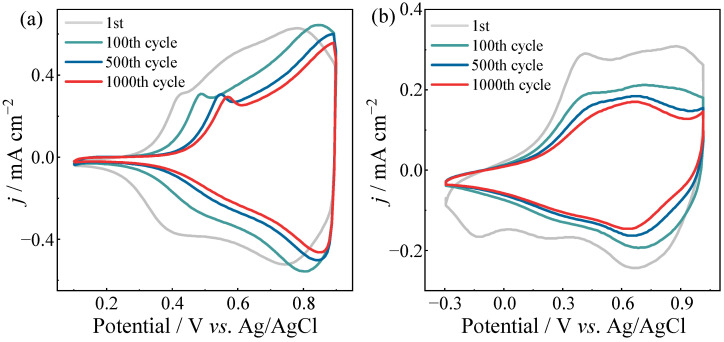
The first, the 100th, the 500th, and the 1000th CV of (**a**) **P4BD-EDOT** and (**b**) **P3BD-EDOT**. Scanning rates at 150 mV s^−1^.

To further investigate the redox stability of these two materials, CV tests were conducted on the polymer films deposited on the working electrode in monomer-free 0.1 M ACN-Bu_4_NPF_6_ at a scan rate of 150 mV s^−1^, as shown in Figure 4. The redox activities of **P4BD-EDOT** remained about 81.3% after 100 cycles in the applied voltage interval (0.1–0.9 V), which was obviously clearly superior to that of **P3BD-EDOT** (69.5% retention after 1000 cycles in the applied voltage interval (−0.3–1.02 V)). After 500 cycles, the **P3BD-EDOT** maintains 62.9% of the initial electroactivity, and the **P4BD-EDOT** remains 56.5% of the initial electroactivity. Whereas in long-time cycles, both of them could maintain long-term redox stability; their electrochemical activity maintains about 55.3% **(P3BD-EDOT)** and 49.2% (**P4BD-EDOT)** of the initial electroactivity after 1000 cycles, respectively. From this, it can be seen that they are promising candidate materials in the electrochromic applications.

### 2.4. FT-IR Spectra

Figure S6 presents the Fourier-transform infrared (FTIR) spectra of the monomers (**4BD-EDOT** and **3BD-EDOT**) and their corresponding polymers (**P4BD-EDOT** and **P3BD-EDOT**). The monomer spectra exhibit characteristic bands in the 3000–2800 cm^−1^ region, corresponding to aliphatic C-H stretching vibrations from the ethylenedioxy groups of EDOT and biphenyl units. Additional spectral features include vibrations associated with C=C bonds within thiophene rings, intramolecular C-C stretching, and C-S bonding (observed near 1074 cm^−1^) with the distinctive vibrational modes of the EDOT ethylene-dioxy rings being particularly prominent. In the polymerized samples (**P4BD-EDOT** and **P3BD-EDOT**), a significant attenuation or complete disappearance of monomer-specific peaks is evident, confirming successful electropolymerization.

### 2.5. Surface Morphology

**Figure 5:**
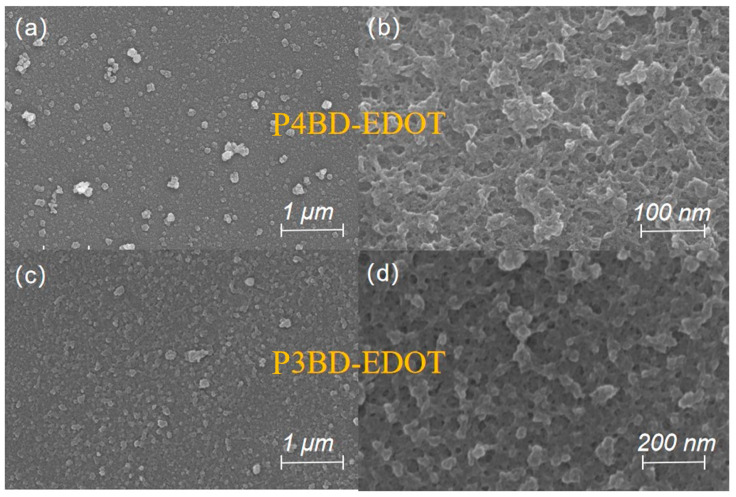
SEM images of **P4BD-EDOT** (**a**,**b**) and **P3BD-EDOT** (**c**,**d**) deposited on the ITO surface.

Figure 5 depicts the SEM images of **P4BD-EDOT** and **P3BD-EDOT**. Low-magnification images (Figure 5a,c) reveal that both polymers form agglomerated particle morphology, which is relatively continuous and compact, a structure that facilitates charge transfer. Higher-magnification images (Figure 5b,d) show that the polymers form a three-dimensional network structure with interstitial pores ranging from 100 to 200 nm. This structure effectively increases the specific surface area of the film in direct contact with the electrolyte, which facilitates the embedding and detachment of electrolyte ions, thus improving the electrochemical activity of the resultant polymer films. In contrast to the compact surface morphology of **P4BD-EDOT**, **P3BD-EDOT** exhibits a well-developed three-dimensional porous architecture. The enhanced surface roughness and interconnected pore network provide optimized pathways for ion transport, which directly translates into accelerated electrochromic switching kinetics. Notably, atomic force microscopy (AFM) analysis reveals distinct morphological characteristics between **P4BD-EDOT** and **P3BD-EDOT** (Figure S7). **P4BD-EDOT** exhibits an interconnected nanoporous architecture with an arithmetic mean roughness (Ra) of 2.40 nm, root-mean-square roughness (Rq) of 3.05 nm, and height variation of 38.0 nm. In contrast, P3BD-EDOT demonstrates phase-separated morphology with prominent domain structures, characterized by significantly elevated Ra (9.12 nm), Rq (12.2 nm), and extended height variation of 81.5 nm, resulting in substantially enhanced surface roughness and specific surface area. These distinct morphological features directly govern the transport efficiency of dopant ions during redox processes, consequently modulating the electrochromic performance of the polymer films.

### 2.6. Spectroelectrochemistry

Spectroelectrochemistry is an efficient method for studying changes in the absorption spectra linked to the CV system for obtaining information about the electronic properties of materials [26,30]. Electrochromic polymers have oxidation properties that produce radical cations (polarons and bi-polarons), resulting in the formation of new electrochromic polymers [31]. This leads to new electronic transitions and changes in absorption bands.

**Figure 6:**
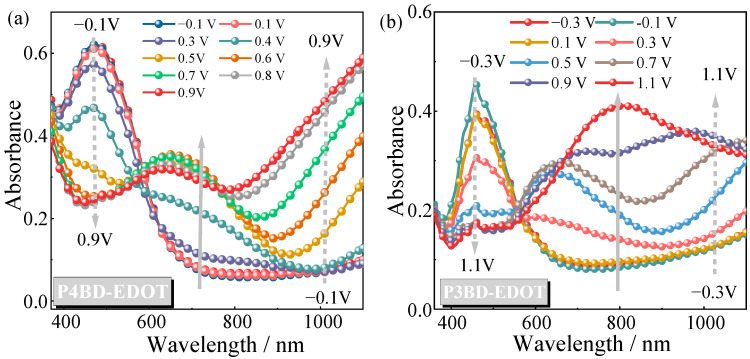
The spectroelectrochemical properties of (**a**) the **P4BD-EDOT** film with applying voltages from −0.1 V to 0.9 V and (**b**) the **P3BD-EDOT** film with applying voltages from −0.3 V to 1.1 V.

The **P4BD-EDOT** and **P3BD-EDOT** films were deposited on the ITO-coated glass, and the spectral changes at different applied potentials were recorded using a UV-Vis spectrophotometer (i.e., in situ spectroelectrochemistry). At first, the **P4BD-EDOT** (−0.1–0.9 V) has different color change characteristics (Figure 6a). The polymer film shows a distinct absorption band at 470 nm, which was due to the π-π* transition in its neutral state. Upon further oxidation, the π-π* transition intensity of **P4BD-EDOT** progressively decreases, concurrent with the emergence and intensification of a new absorption band centered at approximately 770 nm, which is characteristic of polaron formation. As the applied potential is further increased, the polaron band persists, and an additional broad absorption feature develops around 1000 nm, signifying the generation of bipolarons. Concurrently, **P4BD-EDOT** undergoes a distinct color transition from reddish-brown (L* = 56.66, a* = −0.50, b* = 10.72) in the neutral state to blue-purple (L* = 54.46, a* = 8.30, b* = −6.08) in the fully oxidized state, as summarized in Table 1. Meanwhile, the color of **P4BD-EDOT** changed from reddish brown (L* = 56.66, a* = −0.50, b* = 10.72) in the reduced state to blue/purple (L* = 54.46, a* = 8.30, b* = −6.08) in the oxidized state during the oxidation process (Table 1). Compared to **P4BD-EDOT**, **P3BD-EDOT** (−0.3–1.1 V) exhibits a strong absorption at 460 nm (Figure 6b), which was attributed to the π-π* transition of the polymer films [32]. When the potential increases steadily, the intensity of the π-π* transition absorption band decreases, and a new broader band appears, resulting from the polaron band at approximately 710–900 nm [30,31,32,33]. But the formation of a bipolaronic state was not observed. In the oxidation process, **P3BD-EDOT** showed a transparent color (L* = 70.25, a* = 1.31, b* = 8.76) in the neutral state and then changed into light blue (L* = 88.23, a* = −2.51, b* = 3.52) in the oxidized state, accompanied by a reversible color change. According to their absorption edges (**P4BD-EDOT**: 805 nm; **P3BD-EDOT**: 714 nm), their optical band gaps were calculated to be (*E*_g,opt_) 1.54 (**P4BD-EDOT**) and 1.73 eV (**P3BD-EDOT**), respectively. The lower band gap of **P4BD-EDOT** is mainly attributed to the formation of less spatial site resistance at the 4,4′-substitution position, which in turn leads to better optical properties.

### 2.7. Electrochromic Properties of Polymer Films

The kinetics study has been exhaustively investigated as an important parameter to measure the electrochromic performance of the polymer films [34]. Specifically, the change in optical transmittance and the change in response time are measured during the redox process using the interval of maximum absorbance change in the spectroelectrochemical process (Testing condition: MeCN-Bu_4_NPF_6_ (0.1 mol L^−1^). Applying voltage: **P4BD-EDOT** (−0.1~0.9 V), **P3BD-EDOT** (−0.3~1.1 V)).

**Figure 7:**
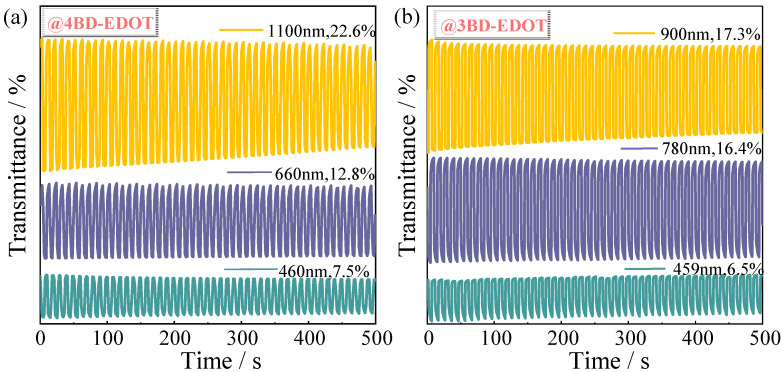
The optical contrast of (**a**) **P4BD-EDOT** and (**b**) **P3BD-EDOT** recorded during double step spectrochronoamperometry for the switching time of 5 s at different wavelengths.

Switching time and optical contrast are two important parameters for evaluating polymers’ electrochromic performance [35] (Figure 7 and Table 2). The optical contrast changes (*ΔT*%) of **P4BD-EDOT** were moderate (7.47% at 460 nm; 12.79% at 660 nm; 22.57% at 1100 nm) (Figure 7a). The selected characteristic wavelengths correspond to distinct spectral regions (visible and near-infrared) where the absorption differential between oxidized and reduced states reaches local maxima. While the response time (95% change in the optical contrast) in the oxidation process was calculated as 1.54 s, 1.67 s, and 0.54 s, the response time in the reduction process was 3.19 s, 3.0 s, and 0.51 s at 460 nm, 660 nm, and 1100 nm, respectively. Likewise, the optical contrast of **P3BD-EDOT** was calculated to be 6.27% at 459 nm, 16.17% at 780 nm, and 16.87% at 900 nm, respectively (Figure 7b). It is interesting to find that **P3BD-EDOT** has a faster response time of 0.21 s in the reduced process compared to 0.33 s in its oxidation process at 459 nm, which was superior to that of **P4BD-EDOT**. The faster response time of P3BD-EDOPT than P4BD-EDOT is attributed to its twisted molecular conformation that generates a porous polymer morphology, which facilitates rapid ion transport. In contrast, the planar structure of P4BD-EDOT favors dense π-π stacking, yielding a compact film that was unfavorable for ion diffusion.

The coloration efficiency is an important index to estimate the electrochromic parameter of polymers, which was evaluated by plotting the correlation between the optical density (ΔOD) and charge density (ΔQ) [36]. The coloration efficiency (*CE*) of **P4BD-EDOT** and **P3BD-EDOT** were calculated as follows: *CE* = ΔOD/ΔQ = log(T_b_/T_c_)/ΔQ, where T_b_ and T_c_ are the transmittances for the bleached and colored states, respectively, as shown in Table 2. The *CE* values of **P4BD-EDOT** were calculated as 189.58 cm^2^ C^−1^, 162 cm^2^ C^−1^, and 189.57 cm^2^ C^−1^ at 460, 660, and 1100 nm, respectively. The *CE* values of **P3BD-EDOT** at 459 nm, 780 nm, and 900 nm are 141.1 cm^2^ C^−1^, 250.4 cm^2^ C^−1^, and 190.4 cm^2^ C^−1^, respectively. Above all, both of them have excellent electrochromic properties in terms of their decent coloration efficiency and fast response time.

Their long-term optical stability was also measured (Figure S8). It was found that the optical contrast of **P4BD-EDOT** showed a slight decrease after 1500 s, whereas the optical activity of **P3BD-EDOT** was totally unstable after 1500 s. This indicates that the degradation of **P4BD-EDOT** is relatively slow, which is consistent with the better electrochemical stability that was obtained from the CV results, providing the necessary conditions for its further application. As summarized in Table S3, a quantitative analysis of EDOT-based polymers demonstrates that the hybrid polymer **P4BD-EDOT** developed in this work exhibits remarkable comprehensive advantages. It achieves an exceptional coloration efficiency of 250.4 cm^2^ C^−1^ at 780 nm in the visible region while maintaining sub-second switching characteristics (≤0.4 s). This particular combination of high coloration efficiency and rapid switching kinetics represents a distinctive advantage rarely found in previously reported EDOT-based polymer systems, underscoring the unique value of our material platform for electrochromic applications.

Furthermore, to rigorously evaluate the long-term operational stability, we designed and fabricated an electrochromic device with a sandwich-type configuration. In this device, **P4BD-EDOT** served as the functional layer, and a PMMA-based gel electrolyte was employed as the ion-conducting medium. The optical performance was systematically investigated under both continuous cycling and open-circuit conditions. As shown in Figure S9, at the characteristic near-infrared wavelength of 1100 nm, the device retained over 75% of its initial optical contrast (*ΔT*%) after a prolonged testing period of 3200 s, corresponding to a decay of less than 25%. This outcome strongly demonstrates the excellent fatigue resistance and reliable long-term operational stability of the **P4BD-EDOT** film in an integrated device configuration, highlighting its promising potential for practical applications. Moreover, the minimal transmittance fluctuation between the neutral and oxidized states further confirms its outstanding optical memory capability and structural integrity.

In summary, the substitution pattern on the biphenyl core—specifically 4,4′-(para-) versus 3,3′-(meta-) linkage—dictates the dihedral angle relative to adjacent conjugated units, ultimately governing the electrochromic performance through distinct structure–property relationships. A smaller dihedral angle, characteristic of the para-substitution, promotes a planar molecular conformation that enhances π-conjugation and optical contrast; however, this molecular arrangement facilitates dense film packing that impedes ion diffusion kinetics. Conversely, the meta-substitution induces a larger dihedral angle while reducing effective conjugation and optical contrast and generates a porous microstructure that significantly accelerates ion transport and enables faster switching dynamics [27,37].

### 2.8. Open Circuit Memory

**Figure 8:**
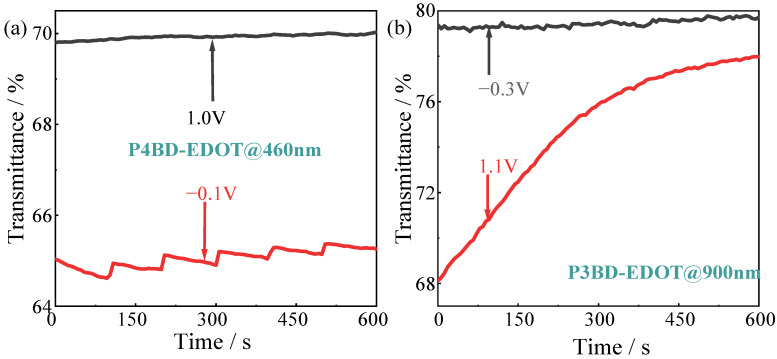
Open circuit memory curves of **P4BD-EDOT** (**a**) monitored at 460 nm and **P3BD-EDOT** (**b**) monitored at 900 nm in ACN-Bu_4_NPF_6_ (0.1 M).

The open-circuit memory is one of the most important parameters for evaluating the performance of electrochromic materials and refers to the ability of a material to maintain electrochromic properties in the absence of applying voltage [38,39]. After complete doping or de-doping in this manner, the optical spectra change In P4BD**-EDOT** and **P3BD-EDOT** at 460 and 900 nm were recorded with every 100 s interval (Figure 8); the applied doping or de-doping voltage was lasted for 2 s. The optical contrast of **P4BD-EDOT** in its oxidation state remains almost unchanged, and in the reduced state, it has just little transmittance changes. In addition, both of them were also tested for the open-circuit memory effect for 3600 s (Figure S10). **P4BD-EDOT** exhibits a half-life (t_1_/_2_) of optical contrast—defined as the time required for the transmittance change (*ΔT*%) to decay to half of its initial value—exceeding 3000 s. In contrast, **P3BD-EDOT** shows rapid degradation of optical contrast at 900 nm, with a transmittance half-life of only about 1200 s. The extended open-circuit memory of **P4BD-EDOT** is attributed to its compact and densely packed film architecture, which effectively stabilizes injected charge carriers and suppresses spontaneous de-doping. This characteristic directly correlates with lower operational energy consumption and extended device shelf life. Conversely, the rapid decay observed in **P3BD-EDOT** underscores a fundamental trade-off between fast switching kinetics and long-term optical bi-stability, highlighting the critical role of molecular and morphological design in optimizing device performance for targeted applications.

## 3. Conclusions

In summary, the polymer films of **P4BD-EDOT** and **P3BD-EDOT** were successfully electrodeposited by electrochemical polymerization of the monomers **4BD-EDOT** and **3BD-EDOT**, applying lower oxidation potentials in Bu_4_NPF_6_-ACN/DCM solution. The electrochemical and spectroelectrochemical behaviors of the homopolymers were investigated with respect to their different monomer structures. Both polymers demonstrated good electrochemical activity, favorable redox stability (55–49% reactivity remained after 1000 cycles), and favorable electrochromic performances. Except for electrochemical performance, altering the EDOT’s replacement position also has an effect on the photoelectric properties of the resulting polymers. **P4BD-EDOT** was reddish brown in the neutral state with two wider absorption peaks at 770 and 1000 nm in the infrared region. In addition, the polymer film also exhibits excellent electrochromic performance with an optical contrast as good as around 23%, a response time less than 0.5 s and higher *CE* of 189.6 cm^2^ C^−1^. While **P3BD-EDOT** displayed higher *CE* (250.4 cm^2^ C^−1^) and lower response time (0.2 s) in the infrared region. All these results indicated that the electrochromic performance of biphenyl-EDOT-based polymers is fundamentally governed by the substitution pattern on the biphenyl unit. The planar architecture of **P4BD-EDOT** (4,4′-substitution) results in superior optical contrast and a reduced bandgap, making it a preferred material for applications requiring high visual distinction. In contrast, the non-planar conformation of P3BD-EDOT (3,3′-substitution) enables faster switching kinetics and enhanced coloration efficiency, advantages derived from its twisted molecular geometry and associated porous morphology. Overall, the combination of high coloration efficiency and faster response time demonstrates both of them are very promising electrochromic materials in the infrared region.

## Figures and Tables

**Table 1:** Electrochemical and spectroelectrochemical data for **P4BD-EDOT** and **P3BD-EDOT**.

Polymer	Redox Stability	λ_max_ (nm)	λ_onset_ (nm)	E_g,opt_ (eV)		Reduction				Oxidation		
						L*	a*	b*		L*	a*	b*
**P4BD-EDOT**	55% (1000 cycles)	457	805	1.54	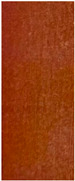	56.66	−0.50	10.72	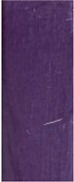	54.46	8.30	−6.08
**P3BD-EDOT**	49% (1000 cycles)	460	714	1.73	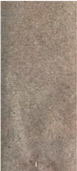	70.25	1.31	8.76	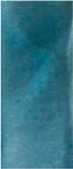	88.23	−2.51	3.52

**Table 2:** Electrochromic properties of **P4BD-EDOT** and **P3BD-EDOT**.

Polymers	Wavelength (nm)	∆*T* (%)	Response Time/s		CE/cm^2^ C^−1^
			Reduction	Oxidation	
**P4BD-EDOT**	460	7.5	3.2	1.5	189.6
	660	12.8	3.0	1.7	162.0
	1100	22.6	0.5	0.5	189.6
**P3BD-EDOT**	459	6.5	0.3	0.2	141.1
	780	16.4	0.3	0.2	250.4
	900	17.3	0.4	0.2	190.4

## Data Availability

The original contributions presented in this study are included in the article/Supplementary Material. Further inquiries can be directed to the corresponding author(s).

## Supplementary Materials

The following supporting information can be downloaded at: https://www.mdpi.com/article/10.3390/nano15211643/s1, Figure S1: Electrochemical polymerization processes and the corresponding polymers **P4BD-EDOT** and **P3BD-EDOT**; Figure S2: ^1^H NMR spectrum of **4BD-EDOT** in CDCl_3_;Figure S3: ^1^H NMR spectrum of **3BD-EDOT** in CDCl_3_; Figure S4: ^13^C NMR spectrum of **4BD-EDOT** in CDCl_3_; Figure S5: ^13^C NMR spectrum of **3BD-EDOT** in CDCl_3_; Figure S6: FT-IR spectra of **4BD-EDOT** and **3BD-EDOT** (a), and corresponding **P4BD-EDOT** and **P3BD-EDOT** (b); Figure S7: Atomic force microscopy (AFM) height images of P4BD-EDOT (a) and P3BD-EDOT (b) films electropolymerized on ITO substrates; Figure S8: Long term optical stability of **P4BD-EDOT** (a) and **P3BD-EDOT** (b).; Figure S9: Long-term Optical Stability of P4BD-EDOT Devices; Figure S10: Long-open circuit memory curves of **P4BD-EDOT** (a) monitored at 460 nm and **P3BD-EDOT** (b) monitored at 900 nm. Table S1: Spectroelectrochemistry of P4BD-EDOT between −0.1 V and 0.9 V (∆E = 0.2 V) and colors variation. Table S2: Spectroelectrochemistry of P3BD-EDOT between −0.3 V and 1.1 V (∆E = 0.2 V) and colors variation. Table S3: Electrochemical and optical performance of polymers. References [40,41,42,43,44,45,46] are cited in the supplementary materials.
