# Supplementary material for: Synthesis, Electrochemistry, and Optoelectronic Properties of Biphenyl-EDOT-Based Electrochromic Polymers
Source: Nanomaterials (Basel). 2025 Oct 28;15(21):1643. doi: 10.3390/nano15211643 (PMC12608673; doi:10.3390/nano15211643)
Supplement: Supplementary file 1 [file nanomaterials-15-01643-s001.zip › nanomaterials-3865305-supplementary.pdf]

Synthesis, Electrochemistry, and Optoelectronic Properties of Biphenyl-EDOT-Based Electrochromic Polymers

Shuanglai Shen [1], Yaoteng Deng [1], Daize Mo [1,*], Mengze Xu [2,*] and Kuirong Deng [1,*]

[1] School of Applied Physics and Materials, Wuyi University, Jiangmen 529020, China;
15171640208@163.com (S.S.); dengyaoteng@163.com (Y.D.)
[2] Department of Biology, Faculty of Arts and Sciences, Beijing Normal University, Zhuhai 519087, China
* Correspondence: modaiz@163.com (D.M.); mengzexu@bnu.edu.cn (M.X.); dengkuir@mail2.sysu.edu.cn (K.D.)

Experimental section

1 Materials

4,4'-dibromo-1,1'-biphenyl, 3,3'-dibromo-1,1'-biphenyl and trans-dichlorobis (triphenyl-phosphine) palladium (II) ($Pd(PPh_3)_2Cl_2$, 98%) were purchased from Shanghai BIDE. *N*,*N*-dimethylformamide (DMF), 3,4-ethylenedioxythiophene (EDOT) and tetrabutylammonium hexafluorophosphate (Bu_4NPF_6) were purchased from Shanghai Energy & Chemical Company. Petroleum ether, methylene chloride (DCM), and acetonitrile (ACN) were obtained came from Tianjin Yongda Chemical Reagent Company. Toluene was purchased from Xilong Company. All these chemicals are used directly without further purification.

2 Synthesis of Monomer

4,4'-bis(2,3-dihydrothieno[3,4-b][1,4]dioxin-5-yl)-1,1'-biphenyl (4BD-EDOT). In a 250 mL single-neck round-bottom flask, a mixture of 4,4'-dibromo-1,1'-biphenyl (1.0 g, 3.20 mmol), 3,4-ethylenedioxythiophene (3.46 g, 8.01 mmol), $Pd(PPh_3)_2Cl_2$ (0.089 g, 0.128 mmol), 80 mL toluene, and 20 mL DMF was heated for 24 h at 120 °C under a nitrogen atmosphere. After cooling to room temperature, saturated salt solution

and dichloromethane were used for extraction. All solvent was removed through decompression concentration, purification was carried out with a silica gel column with V(petroleum ether):V(dichloromethane) = 1:5 used as the eluent. A beige color powder product (0.414 g) was obtained in 50.14% yield. ^{1}H NMR (400 MHz, $CDCl_3$) δ 7.78 (d, J = 8.4 Hz, 4H), 7.62 (d, J = 8.5 Hz, 4H), 6.32 (s, 2H), 4.30 (dtd, J = 26.2, 4.0, 2.5 Hz, 8H). ^{13}C NMR (101 MHz, $CDCl_3$) δ 142.30, 138.67, 138.31, 132.22, 126.95, 126.37, 117.23, 97.74, 64.82, 64.50.

3,3'-bis(2,3-dihydrothieno[3,4-b][1,4]dioxin-5-yl)-1,1'-biphenyl (3BD-EDOT). The method of synthesizing **(3BD-EDOT)** was similar to that of **(4BD-EDOT)** as a white powder (542 mg, yield: 62.19%). ^{1}H NMR (400 MHz, $CDCl_3$) δ 7.94 (s, 2H), 7.71 (d, J = 7.1 Hz, 2H), 7.45 (d, J = 8.0 Hz, 4H), 6.38 (s, 2H), 4.30 (d, J = 23.3 Hz, 8H). ^{13}C NMR (101 MHz, $CDCl_3$) δ 142.26, 141.63, 138.34, 133.57, 129.00, 125.64, 125.12, 125.08, 117.36, 97.81, 64.81, 64.51.

3 Characterization

^{1}H NMR measurements were made on Bruker Avance-400 (400 MHz) using deuterium chloroform ($CDCl_3$) as the solvent. Electrochemical studies were conducted using CHI660E electrochemical workstation. Electron absorption UV-Vis-NIR spectra of the polymer films were obtained on a UV-1900i spectrophotometer. Scanning electron microscopy (SEM) images were obtained on NoVa Nano SEM 430 instrument. Fluorescence data were obtained on a steady-state/transient fluorescence spectrometer (F-4600).

4 Electrochemical tests and polymerization

Electrochemical experiments were measured by using a CHI660E electrochemical workstation. An Ag/AgCl electrode was used as the reference electrode, a platinum wire as the counter electrode, and a glassy carbon electrode (d = 3 mm) as the working electrode. Tetrabutylammonium hexafluorophosphate (Bu_4NPF_6) dissolved in organic solvents (0.1 M) was employed as the supporting electrolyte. Electropolymerization of the monomers (4BD-EDOT and 3BD-EDOT) was performed in DCM with 0.1 M Bu_4NPF_6 at a scan rate of 100 mV s^{-1} for 10 cycles under ambient conditions. At the monomer's neutral potential (-0.1-0.9 V for 4BD-EDOT) and (-0.3-1.1 V for 3BD-EDOT) and oxidation state (approximately 0.9 V for 4BD-EDOT) and (1.1 V for 3BD-EDOT), colored polymer films were electrodeposited on glassy carbon electrode. Cyclic voltammograms of the polymer films were obtained using the same electrode setup in the monomer-free ACN solution containing 0.1 M Bu_4NPF_6. For spectroelectrochemical experiments, the polymer films were electrodeposited in the same fashion on an ITO-coated glass electrode and their UV-Vis-NIR spectra at different applied potentials were recorded.

The UV-visible absorption spectra were characterized using a potential-step method. Polymer films were initially formed by potentiostatic deposition at specific potentials for 30 seconds. For P4BD-EDOT, spectroelectrochemical measurements were conducted over a potential range of -0.1 V to +0.9 V (vs. Ag/AgCl) with 0.1 V increments, while P3BD-EDOT was characterized from -0.3 V to +1.1 V (vs. Ag/AgCl) using 0.2 V steps. At each potential, the system was allowed to equilibrate for 20 seconds to establish electrochemical steady-state conditions before acquiring stable

absorption spectra using a UV-1900i spectrophotometer.

Since the charge stored by the material (capacitance, C) is directly proportional to the area enclosed by the CV curve, we used the decay of the CV area as a metric for performance degradation. The percentage retention after N cycles was calculated according to:

Retention (%) = (A_N / A_1) × 100%

Here, A_1 denotes the initial integrated area of the CV curve at the 1st cycle, serving as the baseline (100%). A_N represents the integrated area of the CV curve recorded at the N-th cycle.

Optical absorption measurements provide energy difference values between the previously described band edges. The $E_{g,opt}$ of all precursors and corresponding polymers was determined from their onset absorption (λ_{onset}) from UV-vis spectra with the following equation:

$$E_{g,opt} = 1241 / \lambda_{onset} (\mathrm{eV})$$

5 Spectroelectrochemical and electrochromic studies

Spectroelectrochemical and electrochromic studies were tested on a UV-1900i spectrophotometer under control of an electrochemical workstation (CHI660E). An Ag/AgCl electrode as the reference electrode, indium tin oxide (ITO)-coated glass slide as the working electrode, and a Pt wire as the counter electrode in a transparent cuvette. These characterizations were all performed in ACN-Bu_4NPF_6 (0.1 M) electrolytes.

The potentials were alternated between the reduced and oxidized states with a residence time of 5 s. The optical contrast at the specific wavelength (λ) was determined

by ΔT% values of polymer films, using the following equation:

$$\Delta T = |T_{ox} - T_{red}|$$

The coloration efficiency (*CE*) is defined as the relation between the injected/ejected charge as a function of electrode area (Q_d) and the change in optical density (ΔOD) at the specific wavelength (λ) of the sample as illustrated by the following equation:

$$CE = \Delta OD/Q_d$$

$$\Delta \mathrm{OD} = \log(\mathrm{T_{ox}}/\ \mathrm{T_{red}})$$

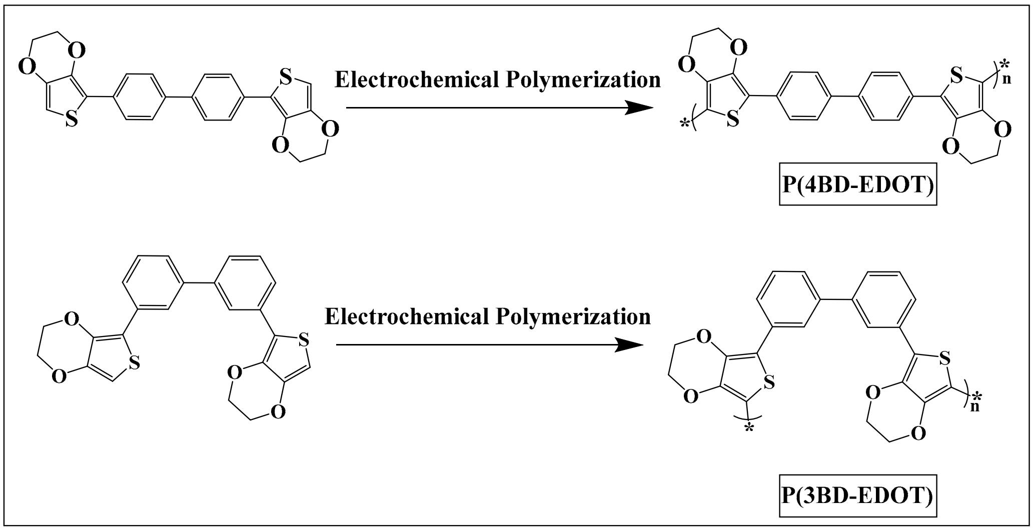

Figure S1. Electrochemical polymerization processes and the corresponding polymers **P4BD-EDOT** and **P3BD-EDOT**.

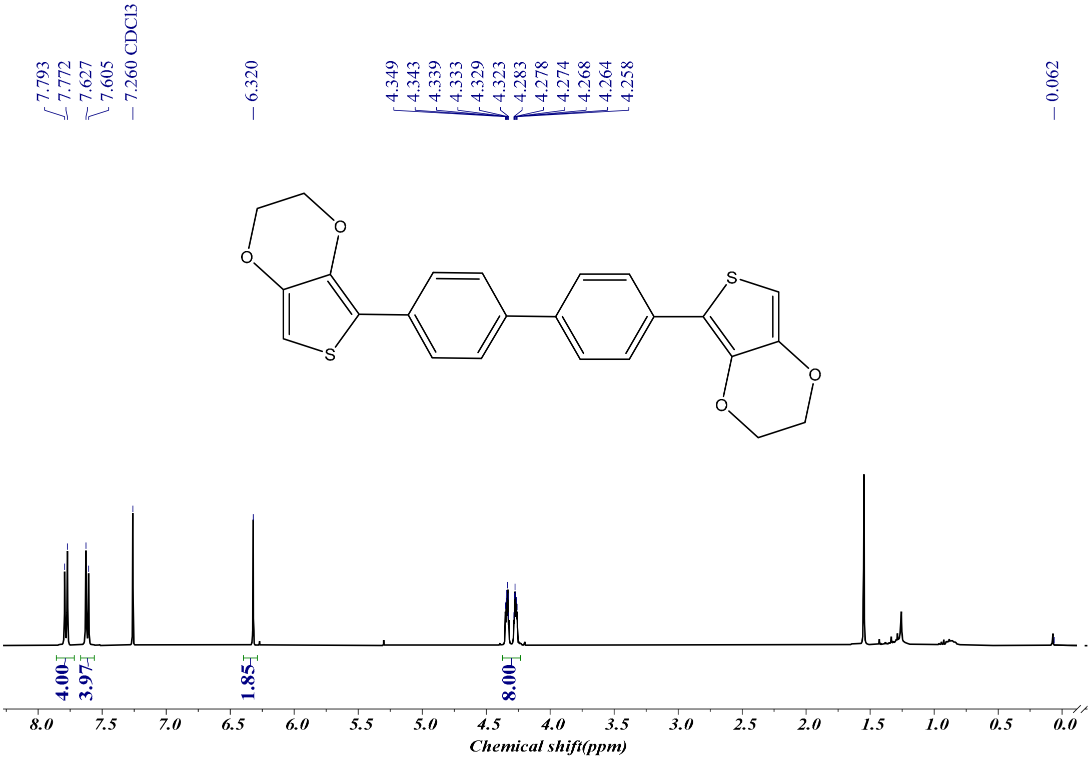

Figure S2. ^{1}H NMR spectrum of **4BD-EDOT** in $CDCl_3$.

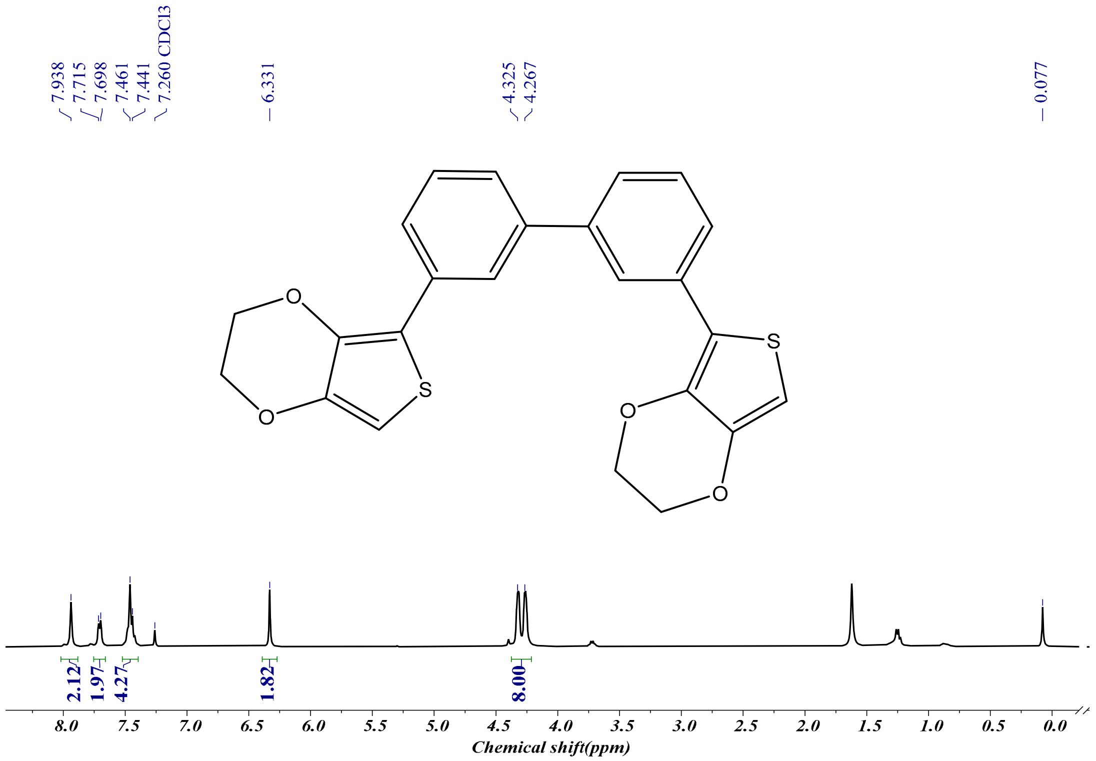

Figure S3. ^{1}H NMR spectrum of **3BD-EDOT** in $CDCl_3$.

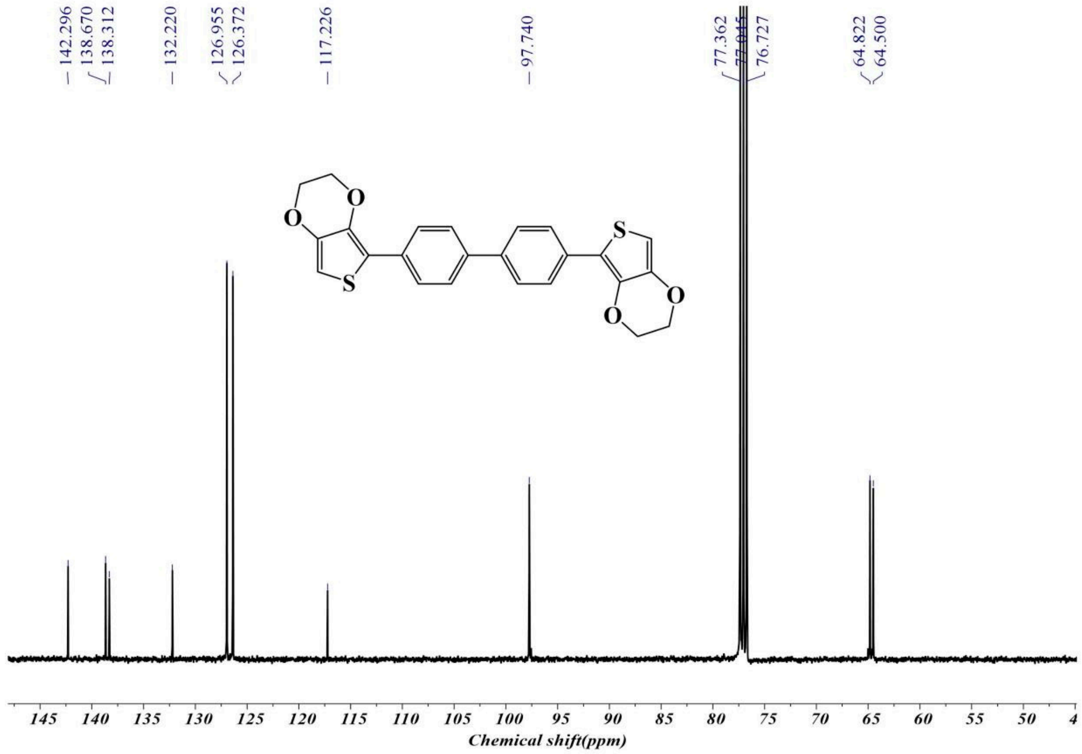

Figure S4. ^{13}C NMR spectrum of **4BD-EDOT** in $CDCl_3$.

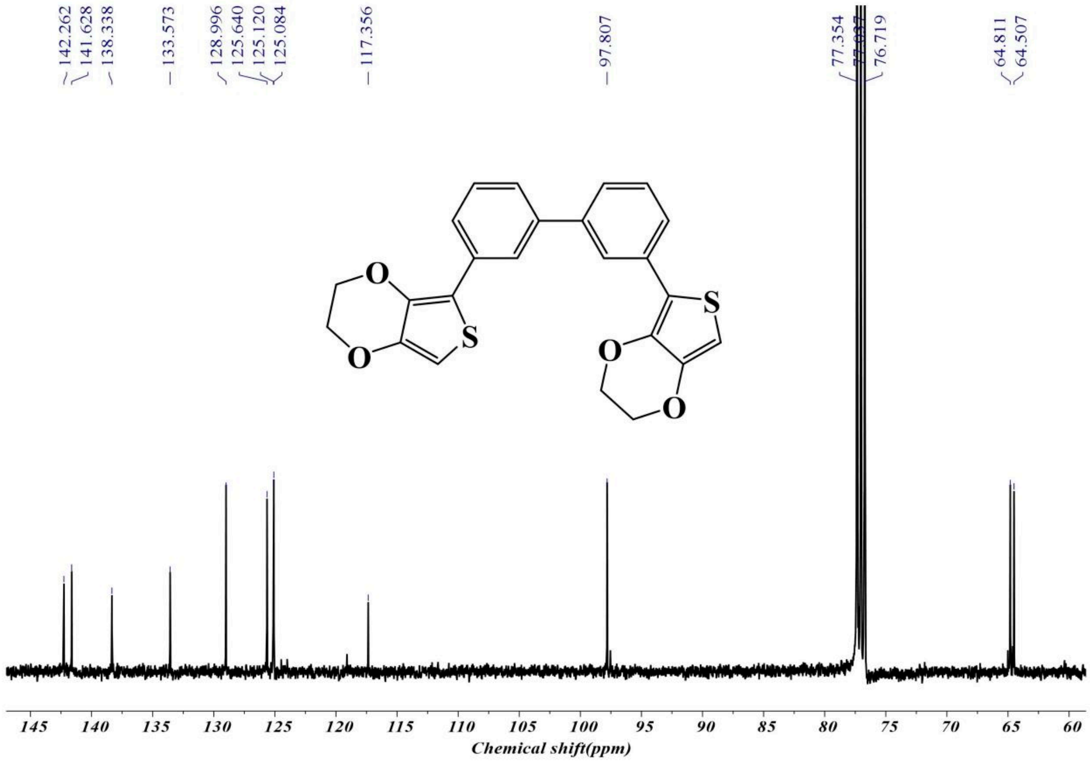

Figure S5. ^{13}C NMR spectrum of **3BD-EDOT** in $CDCl_3$.

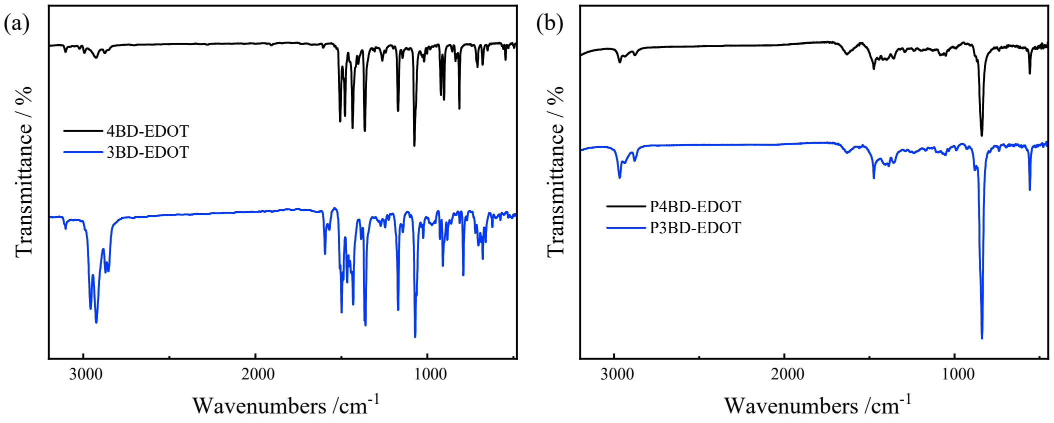

Figure S6. FT-IR spectra of **4BD-EDOT** and **3BD-EDOT** (a), and corresponding **P4BD-EDOT** and **P3BD-EDOT** (b).

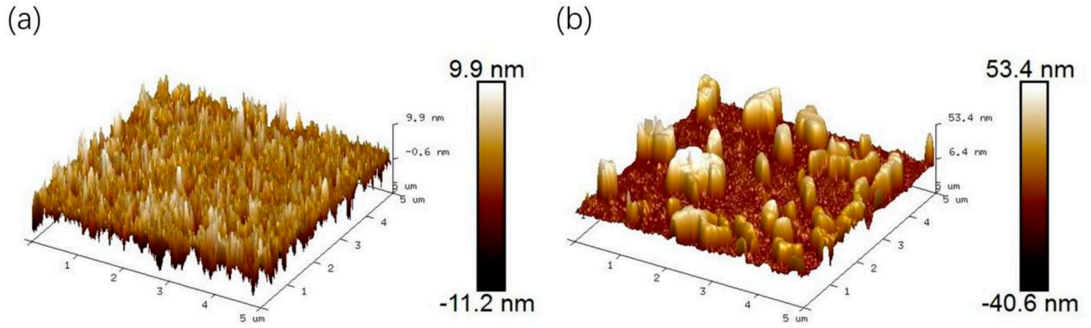

Figure S7. Atomic force microscopy (AFM) height images of P4BD-EDOT (a) and P3BD-EDOT (b) films electropolymerized on ITO substrates.

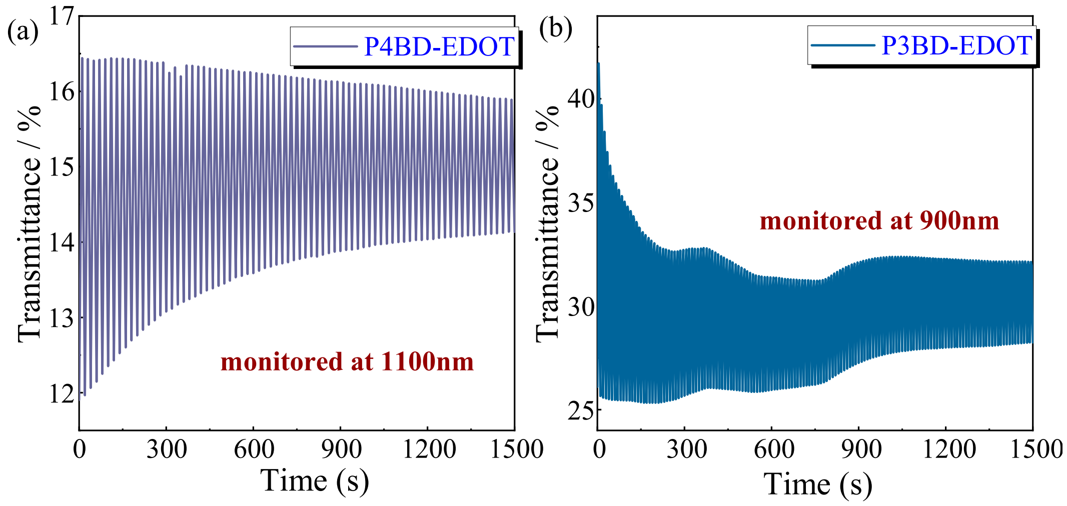

Figure S8. Long term optical stability of **P4BD-EDOT** (a) and **P3BD-EDOT** (b).

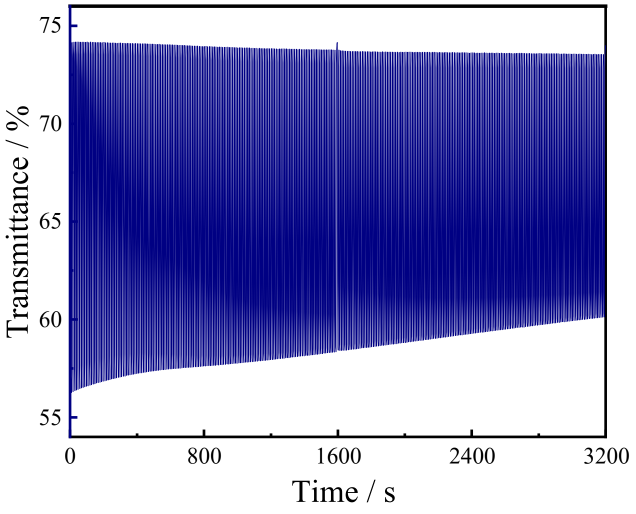

Figure S9. Long-term Optical Stability of P4BD-EDOT Devices.

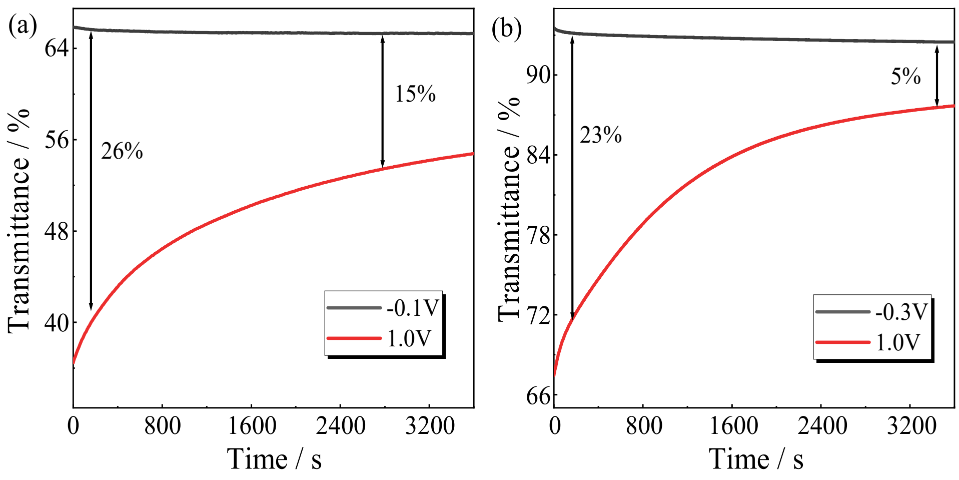

Figure S10. Long-open circuit memory curves of **P4BD-EDOT** (a) monitored at 460 nm and **P3BD-EDOT** (b) monitored at 900 nm.

Table S1 Spectroelectrochemistry of **P4BD-EDOT** between -0.1 V and 0.9 V (ΔE = 0.2 V) and colors variation.

Compound	Potential (V)	L*	a*	b*	Color
P4BD-EDOT	-0.1	28.66	39.77	-1.79	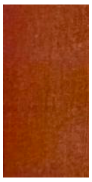
	0.1	28.13	40.01	-3.39	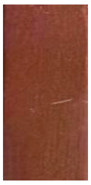
	0.3	28.02	39.41	-5.94	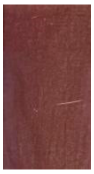
	0.5	25.54	40.12	-21.45	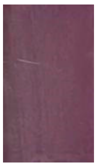
	0.7	23.55	43.52	-34.92	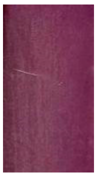
	0.9	22.53	44.76	-36.97	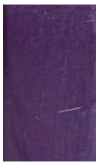

Table S2 Spectroelectrochemistry of **P3BD-EDOT** between -0.3 V and 1.1 V (ΔE = 0.2 V) and colors variation.

Compound	Potential (V)	L*	a*	b*	Color
P3BD-EDOT	-0.3	70.25	1.31	8.76	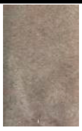
	-0.1	89.15	2.34	8.63	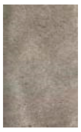
	0.1	89.11	1.61	8.02	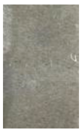
	0.3	88.89	0.02	6.17	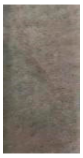
	0.5	88.03	-2.22	4.07	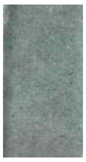
	0.7	88.23	-2.51	3.52	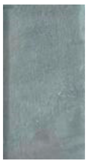
	0.9	88.41	-1.74	3.46	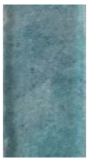
	1.1	88.05	-1.13	3.07	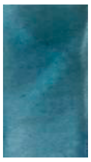

Table S3 Electrochemical and optical performance of polymers.

Polymer	ΔT/%	Response time / s		$CE/C^{-1}\ cm^2$	Ref
		Reduction	Oxidation		
P(1Cl)	10 (376 nm)	0.6	2.2	195.6	[1]
	24 (1100 nm)	0.7	3.96	152.6	
P(2Cl)	4 (460 nm)	0.6	1.74	49.2	[1]
	1 (710 nm)	0.4	0.72	23.8	
	10.5 (1100 nm)	0.8	1.32	136.9	
P(F-BT-EDOT)	33 (1100 nm)	0.9	1.32	196.3	[2]
	16 (723 nm)	0.7	1.14	109.5	
	19 (471 nm)	1.1	1.6	124.7	
P(FF-EDOT)	27.90(1100nm)	0.62	0.97	122.80	[3]
	30.82(889nm)	1.76	2.00	142.12	
	19.48(455nm)	1.89	1.84	119.00	
P(Se-EDOT)	29.05(1100nm)	0.4	0.515	270.13	[4]
	2.93(589nm)	0.64	0.265	53.26	
	3.79(420nm)	0.79	0.805	78.70	
P(Se-Th-EDOT)	31(1100nm)	0.43	0.77	160.08	[4]
	33.32(850nm)	0.44	0.805	140.38	
	16.4(408nm)	0.53	0.895	109.31	
P(FF-EDOT)	28.55(1100nm)	0.35	0.55	163.50	[5]
	19.77(959nm)	0.33	0.49	134.94	
	20.91(487nm)	0.58	0.63	259.68	
PEDOT	54%	--	--	137	[6-7]
P4BD-EDOT	7.5(460nm)	3.2	1.5	189.6	this work
	12.8(660nm)	3.0	1.7	162.0	
	22.6(1100nm)	0.5	0.5	189.6	

P3BD-EDOT	6.5(459nm)	0.3	0.2	141.1	this work
	16.4(780nm)	0.3	0.2	250.4	
	17.3(900nm)	0.4	0.2	190.4	

[1] Daize Mo, Tong, Kaiwen Lin, Chlorinated benzothiadiazole-based donor-acceptor polymers with tunable optoelectronic performances, Electrochim. Acta 473 (2024) 143506.

[2] D.Z. Mo, T. Tong, PJ. Chao, et al. Effects of polymer precursor conjugation length on the optoelectronic properties of fluorinated benzothiadiazole-based D–A systems[J]. New Journal of Chemistry, 48(2024) 7590-7598.

[3] Z. Ren, D.Z. Mo, S. Wang, T. Tong, K.R. Deng, P. Chao, Effects of fluorine atom numbers on electrochromic properties of the benzothiadiazole-based DA polymers. Polymer, 312(2024)127655.

[4] D.Z. Mo, Z. Ren, K.R. Deng, P.J. Chao. Difluorinated benzoselenadiazole: A new promising electron withdrawing acceptor unit for building efficient DA type electrochromic polymers[J]. Polymer, 319(2025)128068.

[5] Z. Ren, J. Zhou, D. Mo, et al. Difluorinated benzothiadiazole based donor-acceptor electrochromic polymers with tunable optoelectronic properties by varying the thiophene donor units[J], Synth. Met. (2025)117946.

[6] M. Li, Sheynin. Y, Patra. A and Bendikov. M, Tuning the electrochromic properties of poly (alkyl-3, 4-ethylenedioxyselenophenes) having high contrast ratio and coloration efficiency. Chem. Mater, 21 (2009), 2482-2488.

[7] Sonmez. G, Meng. H and Wudl. F, Organic polymeric electrochromic devices: polychromism with very high coloration efficiency. Chem. Mater, 16(2004), 574-580.
